# Simple Millimeter Wave Identification System Based on 60 GHz Van Atta Arrays

**DOI:** 10.3390/s22249809

**Published:** 2022-12-14

**Authors:** Kamil Trzebiatowski, Mateusz Rzymowski, Lukasz Kulas, Krzysztof Nyka

**Affiliations:** Department of Microwave and Antenna Engineering, Faculty of Electronics, Telecommunications and Informatics, Gdansk University of Technology, Narutowicza 11/12, 80-233 Gdansk, Poland

**Keywords:** chipless radio-frequency identification (RFID), tag localization, millimeter wave identification (MMID), radar cross-section (RCS), retrodirective array, Van Atta array

## Abstract

The paper presents a proof-of-concept of a millimeter-wave identification system based on Van Atta array tags in the 60 GHz band. For interrogation of the tags, a vector network analyzer and a measurement transceiver were employed in alternative test configurations. The design, fabrication and measurements of co- and cross-polarized Van Atta arrays are presented in the paper. They can be treated as simple chipless RFID tags with frequency-response-based identification. Tags with various resonance frequencies are designed by scaling an optimized base model. The designed 57–67 GHz co-polarized and cross-polarized tags have small dimensions of approximately 23 mm × 21 mm and 40 mm × 25 mm, and they exhibit radar cross-section (RCS) levels of −16 dBsm and −21 dBsm, respectively. Owing to the retrodirective properties of Van Atta arrays, the RCS can be maintained at a high level within a broad range of angles of incidence. The system was validated in an anechoic chamber where the spectral responses of all the manufactured tags can be clearly distinguished from the environment, enabling their identification. Tests in a reflective environment were also performed, and they have shown that only the cross-polarized tags could be detected and identified in the presence of reflections from the tags’ surroundings.

## 1. Introduction

The V-band (50–75 GHz), and especially the 60 GHz Industrial, Scientific and Medical (ISM) band, are considered for the implementation of millimeter wave identification (MMID) systems [1]. These systems are an extension of the conventional radio frequency identification systems (RFID) into the millimeter wave range, i.e., frequencies above 20 GHz. The main difficulty hindering the development of the MMID systems is the high cost of RFID tags [1,2,3], which is influenced primarily by the integrated circuit (IC) price. One of the proposed solutions to reduce costs is to employ chipless RFID tags, which do not contain ICs [2,4]. These tags encode data in their reflection responses, modifying the parameters of the backscattered wave and relying only on passive microwave circuits. They can be realized by employing techniques such as time-domain reflectometry, frequency encoding (spectral signatures) or amplitude/phase backscatter modulation [5].

**Figure 1:**
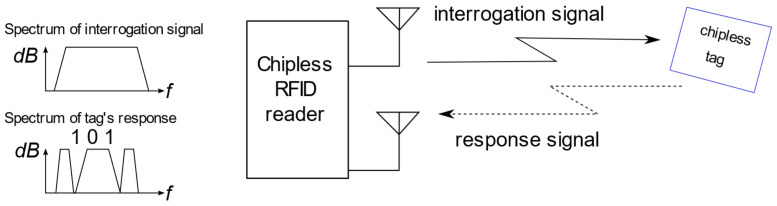
Principle of the frequency-encoding chipless RFID system.

The schematic of a typical chipless RFID system based on tags with spectral encoding [2,6,7] is presented in Figure 1. The system is composed of a reader with transmitting (Tx) and receiving (Rx) antennas and an interrogated chipless tag. The reader emits an interrogation signal which is then modified and reflected back by the tag. The response signal is received by the antenna and the information is extracted by the reader.

In the literature, there are multiple examples of frequency-encoding tags realized in printed-circuit-board technology (PCB) [5,8,9,10]. Such tags comprise two high-gain antennas (typically orthogonally polarized) and a line connecting them that is coupled with resonators performing the function of encoding the information. The data is encoded as the presence or absence of resonance peaks in the frequency response of the reflected wave, as presented in Figure 1. A wideband system is required to accurately read the information encoded in this type of tag, as all resonance peaks have to be extracted from the response. Due to the small antenna gain and small corresponding radar-cross section (RCS), especially in the millimeter-wave range, these tags suffer from relatively short read distances and narrow read angle ranges, which are only within near to perpendicular incidence of the interrogating signal [2].

These disadvantages can be mitigated by the use of Van Atta arrays, which allow for increasing the strength of the signal reflection from the tag (increasing its RCS), and at the same time, they significantly increase the angular reading range. Thanks to this retrodirective property, the precise alignment of the main axis of the tag with the main axis of the reader is not necessary. Unfortunately, Van Atta arrays have large electrical dimensions, and so using them in RFID tags becomes worth considering primarily in the millimeter wave bands. With the very small wavelength at 60 GHz, the tags can have reasonably small sizes [11]. Another advantage of such high operating frequencies is that high gain antennas can be used in the readers, and thus, the angular reading resolution can be significantly enhanced [12], allowing a precise angular scanning for the localization of the tags.

The operation of millimeter-wave Van Atta array-based tags was previously presented in the Ka-band, and it was demonstrated that they can be used in a long-range RFID system if coupled with high-gain amplifiers and high-gain transmitting and receiving antennas. The tags were detectable in a reflective environment with distances between the tag and the reader of up to 30 m [13]. The complex response, using both amplitude and the phase of the reflection, was used to detect and identify the tag. It was not presented, however, if it was possible to reliably detect the tags with only the amplitude information of the received signal. Millimeter-wave Van Atta tags working in the V-band (60 GHz) were recently investigated in [11], but only the basic parameters of the co-polarized and cross-polarized tags were numerically compared, and their realization was neither reported nor experimentally verified.

In this paper, a proof-of-concept for a simple MMID system that can detect and identify 60 GHz-band chipless tags based on Van Atta arrays is presented. According to the authors’ knowledge, this is the first time the operation of a 60 GHz MMID system, which relies solely on the amplitude frequency response recorded by the reader, is demonstrated in a series of measurements. The manufactured co-polarized and cross-polarized tags with different values for the resonance frequencies, being a distinguishing identification parameter, were manufactured using PCB technology and their basic characteristics were measured in an anechoic chamber. The reading and identification method based only on the amplitude frequency response is shown, validating the numerical simulations and verifying the tags’ operation and performance in a real-life reflective indoor environment. A simple transceiver system without high-gain amplifiers is shown to be sufficient to properly read and identify the tags at a distance of 3 m, which allows for the consideration of the proposed solution in applications relying on inexpensive hand-held MMID readers in the future. Additionally, the employed Van Atta array principle makes it possible to read the tags in a wide angular range. Such a functionality opens up new application areas as such chipless and batteryless tags can be easily integrated with road or factory infrastructure to support autonomous vehicle navigation.

## 2. Van Atta Array Design

**Figure 2:**
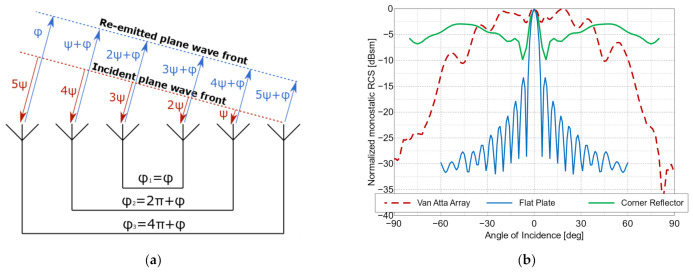
The Van Atta array: (**a**) schematic of the operation principle; (**b**) comparison of the RCS values of a Van Atta array (20 mm × 18 mm), those of a flat metal plate (23 mm × 29 mm), and those of a corner reflector (12 mm × 12 mm × 12 mm), all at a frequency of 62 GHz.

Citing the original L. C. Van Atta patent, a Van Atta array is “a passive electromagnetic device for receiving an incident electromagnetic wave and transmitting this wave back in the direction from whence it came, comprising a linear or two-dimensional array of no less than four antennas, interconnected by electromagnetic paths of equal length between antennas of the array that are symmetrically disposed about a geometrical center of the array” [14,15]. The basic principle of retrodirectivity exhibited by Van Atta arrays is shown in Figure 2a. An incident plane wave arriving from an arbitrary angle is received by the array’s antennas, resulting in a relative phase shift (ψ) of signals excited in the adjacent antenna ports. For each port, the received signals then pass through interconnecting lines of electrical length (2*πn* + *φ* (*n =* 0, 1, 2, …)) to their corresponding transmitting radiators, and they are re-radiated with the same relative phase shift (ψ). The waves from all the radiators meet in phase at the plane that is parallel to the wave-front of the incident wave, which means that the wave is re-emitted in the same direction that it arrived from.

The re-radiated wave can be of the same polarization as the incident wave in the case of a co-polarized array. In the case of a cross-polarized array, the polarization of the re-emitted wave is orthogonal to the received wave. The key feature of Van Atta arrays is their wide angular range of almost uniform reflection characteristics. This property can be seen in Figure 2b, where the RCS values of an example co-polarized Van Atta array of 20 mm × 18 mm size are compared to the RCS values of a flat plate with dimensions of 23 mm × 29 mm and a 3D corner reflector with a side length of 12 mm. The Van Atta array’s RCS characteristics are more uniform in the −45° to 45° range of interrogation angles compared to the standard corner reflector, but this falls off quickly for the angles further from the main axis, whereas the corner reflector has a high RCS value for the angles close to the parallel incidence (−90° and 90°). Because the strictly defined phase shifts are realized by transmission lines, Van Atta arrays maintain their retrodirective properties only in a narrow band near the resonance frequency. Although usually considered a drawback, this feature can be turned into an advantage, as it provides the means to construct a simple chipless RFID tag out of a single Van Atta array, with a unique resonance frequency serving as the tag’s identifier. The design of both co- and cross-polarized Van Atta arrays is presented in the paper, and three tags of each type were designed with different resonance frequencies as their distinguishing features. The tags were manufactured using PCB technology on a 0.127 mm thick ROGERS RT/duroid 5880 substrate (ε_r_ = 2.20 and tanδ = 0.001).

### 2.1. Co-Polarized Array

**Figure 3:**
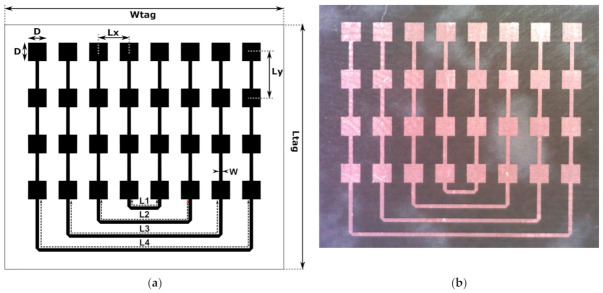
The co-polarized Van Atta arrays: (**a**) design and dimensions, and (**b**) photograph of the manufactured tag.

The designed co-polarized Van Atta arrays consist of *N* (here, *N* = 4) pairs of interconnected linear arrays in the vertical direction, as shown in Figure 3. A single array consists of four series-fed square microstrip patches and is equivalent to one antenna, as presented in Figure 2a. The patch sizes *D* and spacing *Ly* are selected to achieve a single array’s input impedance of approximately 50 Ohm at the resonance frequency. The arrays are spaced by *Lx* from each other and are interconnected by microstrip lines of width *W* and length *Ln* (*n* = 1, *…*, *N*). The lengths of the successive interconnecting lines differ in multiples of the guided wavelength *λ_g_* such that all the arrays are fed in-phase.

The tag dimensions were obtained by manual tuning and automatic optimization, for which the Global Response Surface Method available in the Altair Feko environment was used, and they are presented in Table 1. The goal was to maximize the monostatic radar cross-section level over the −60° to 60° angle range of the incident wave direction. Optimization was performed for the 62 GHz Van Atta array with *N* = 4 pairs of interconnected linear arrays. All the dimensions (except the line width) of the array were formulated in multiples of the wavelength, which enabled the quick design of the 57 GHz and 67 GHz arrays.

### 2.2. Cross-Polarized Array

**Figure 4:**
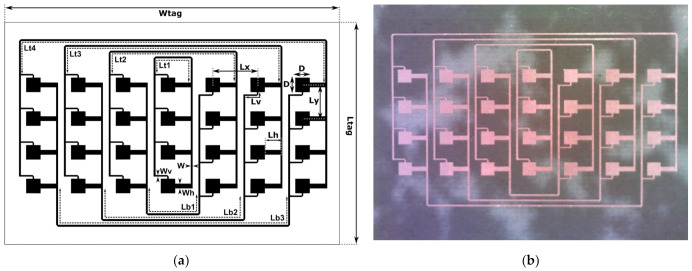
The cross-polarized Van Atta array: (**a**) design and dimensions, and (**b**) photograph of the manufactured tag.

The design of the cross-polarized array is presented in Figure 4. Similar to the co-polarized design, the Van Atta array comprises *N* pairs (here, *N* = 4) of microstrip linear vertical arrays spaced by *Lx* from each other, but the central self-connected array is counted as one pair. A single array consists of four microstrip patches with a distance *Ly* between them and a *D* side length. The patches can be fed from two orthogonal sides, which allows for a dual-polarized operation. They are connected to the main feed line of width *W* (for both polarization cases), with short feeding lines of widths *Wh* and *Wv*, and lengths *Lh* and *Lv* of approximately *λ_g_*/2. As a result, the array, as seen from a single polarization port, functions similarly to the series feed used in the co-polarized tag, with the difference being that it has two separate polarization ports that can be used to excite two orthogonal modes. The interconnecting microstrip lines connect these ports in such a way that the polarization of the re-emitted electromagnetic wave is changed to orthogonal. The tag dimensions are presented in Table 2.

There are *N* lines interconnecting the arrays from the top with lengths *Ltn* and (*N*−1) lines from below with lengths *Lbn*. The patches on the right side have vertical feed ports at the bottom edge, whereas the patches on the left side have vertical feed ports at the top edge. This means that they have an additional phase offset of 180°. To compensate for this effect and to ensure that all arrays are excited in-phase, the lengths *Lbn* are increased by approximately one half-wavelength (0.45 *λ_g_*) compared to the lengths *Ltn*.

## 3. Measurement Setups

For the experimental verification of the proposed solutions, two measurements setups were prepared: one in an anechoic chamber for the RCS measurements of the tags and the other in a realistic indoor reflective environment for the verification of the proposed proof-of-concept MMID. The second, in particular, was used to demonstrate how the cross-polarization tag can suppress background reflection that would affect the readability of the tags.

### 3.1. Anechoic Chamber Measurements

**Figure 5:**
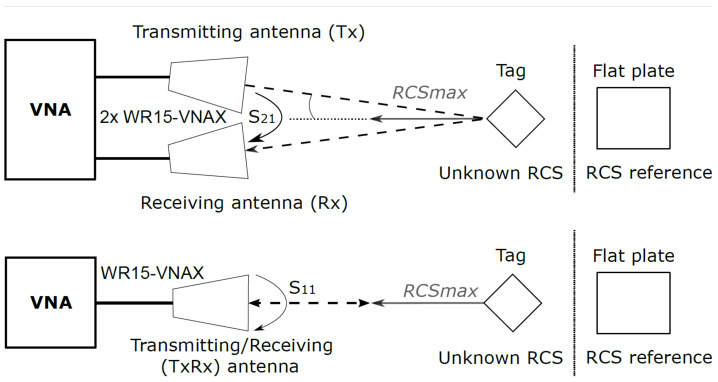
Schematic of the measurement setup in the anechoic chamber.

**Figure 6:**
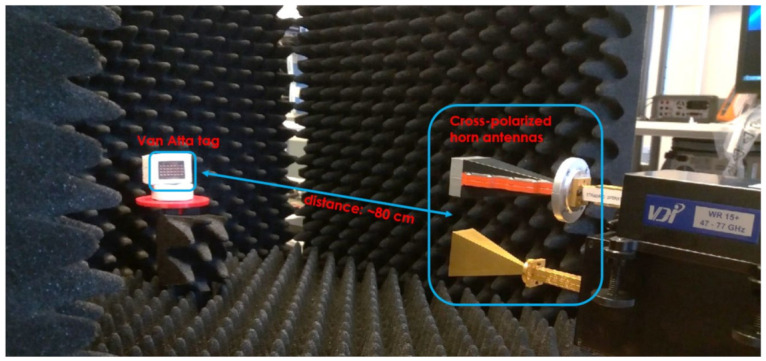
The measurement setup in the anechoic chamber.

The first batch of tests was carried out in a millimeter-wave anechoic chamber with a vector network analyzer (VNA) ZVA50 (Rohde and Schwarz, Munich, Germany) and millimeter-wave extension modules VDI WR15-VNAX (Virginia Diodes Inc., Charlottesville, VA, USA), both of which were added to perform the measurements in the 50–75 GHz band. The output power of the VDI transmitting head was approximately 13 dBm [16]. The measurement schematic is depicted in Figure 5. Two 24 dBi horn interrogating antennas placed very close to each other, as seen in Figure 6, were connected to the VDI transmitting (Tx) and receiving (Rx) heads in such a way that the antennas had orthogonal polarizations. The tags under test were placed on a turntable for the incidence angle sweeping, with 2° steps, at a distance of approximately 0.8 m from the horn antennas. The VNA was used to measure the RCS characteristics of the tags in the function of signal frequency and the angle of incident wave in two configurations. For the co-polar measurements, only the Tx antenna was used, and the RCS was derived from the reflection coefficient (S11) measurement with added time-domain gating, which eliminated the influence of mismatches at the antenna port [17]. The cross-polarized tags were measured in a configuration with both Tx and Rx antennas, where the transmission coefficient (S21) was interpreted as S11 in co-polar measurements. The measurements were calibrated using a flat rectangular metal plate of known size, the RCS of which was numerically calculated and used as a reference [17,18].

### 3.2. Realistic Environment Tests

**Figure 7:**
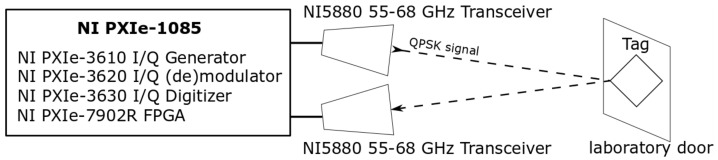
Schematic of the measurement setup in the indoor environment.

**Figure 8:**
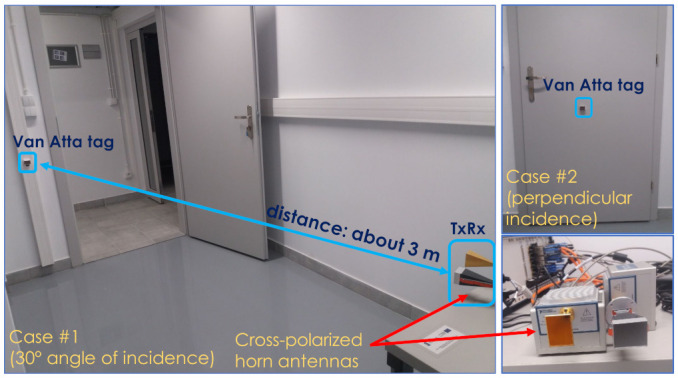
The measurement setup in the indoor environment.

To verify the proposed proof-of-concept MMID system, a second series of experiments was carried out in a realistic reflective environment, i.e., a laboratory room without absorbers suppressing the reflections from the surroundings. In this environment, tests of the reading of the co-polarized and cross-polarized tags at a distance of approximately 3 m were carried out. The tags were placed on a wooden laboratory door. As an interrogator, a National Instruments (NI) Millimeter Wave Transceiver System using NI5880 55–68 GHz heads was used, with the same horn antennas as in the measurements in the anechoic chamber. The experiment setup is depicted in Figure 7 and Figure 8. The transmission measurements with a narrow-band QPSK-modulated signal of swept carrier frequencies from 55 GHz to 68 GHz were performed, and the received signal level was recorded, which was proportional to the RCS of the object under test. The experiments were performed for the three cross-polarized tags having different resonant frequencies and for one co-polarized tag.

## 4. Results and Discussion

### 4.1. Anechoic Chamber Results

**Figure 9:**
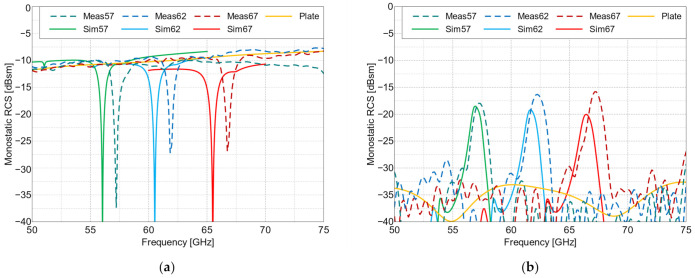
RCS values of the co-polarized arrays and the flat plate of the same size (Plate) vs. frequency, at the following angles of incidence: (**a**) 0° and (**b**) 30°. Meas—measured results and Sim—simulated results. The resonance frequencies of the three manufactured Van Atta tags (in GHz) are 57, 62 and 67.

**Figure 10:**
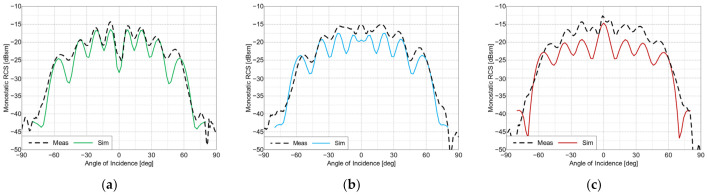
RCS values of the co-polarized arrays vs. angle of incidence, at frequencies of: (**a**) 57 GHz, (**b**) 62 GHz and (**c**) 67 GHz.

**Figure 11:**
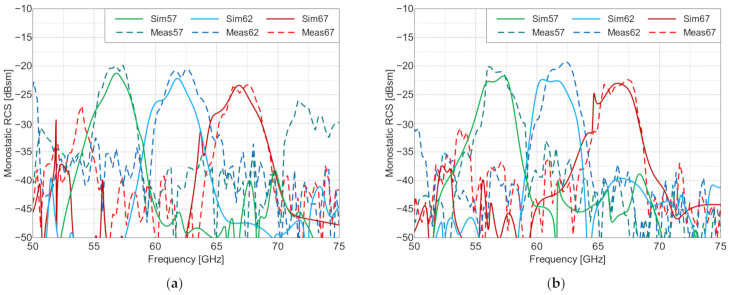
RCS values of the cross-polarized arrays vs. frequency, at angles of incidence of: (**a**) 0° and (**b**) 30°. Meas—measured results and Sim—simulated results. The resonance frequencies of the three manufactured Van Atta tags (in GHz) are 57, 62 and 67.

**Figure 12:**
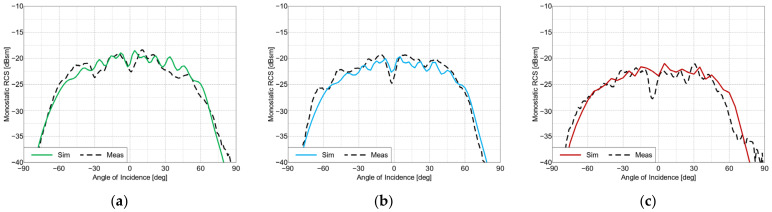
RCS values of the cross-polarized arrays, at frequencies of: (**a**) 57 GHz, (**b**) 62 GHz and (**c**) 67 GHz.

The measured frequency characteristics for the co-polarized tag arrays are shown in Figure 9 and the angular characteristics are presented in Figure 10. For the cross-polarized arrays, these same characteristics are shown in Figure 11 and Figure 12, respectively. To verify the performance of the tags at the perpendicular and oblique incidence of the interrogating waves, the frequency characteristics were measured at incidence angles of 0° and 30°.

The measurement results from the anechoic chamber were compared with the simulations performed in Altair Feko 2021. The basic parameters of the tags are summarized and compared in Table 3. Table 4 contains a comparison of the presented tags to state-of-the-art chipless RFID tags.

The proposed co- and cross-polarized cases tags are easily distinguishable from each other in the frequency domain. The co-polarized tags have narrower frequency responses and higher average RCS levels compared to those of the cross-polarized tags, but the cross-polarized tags can be read from a wider range of angles.

The frequency characteristics of the co-polarized arrays at an incidence angle of 0° is notably different from the other measured frequency characteristics. The reflection over the entire frequency range is dominated by the co-polarized reflections from the tag’s ground plane, with one valley at the resonance frequency caused by the destructive interference of the reflected and re-emitted waves. This effect can be seen in Figure 9, which, among other things, presents a comparison of the RCS values of the co-polarized arrays with the RCS value of a flat metal plate of the same size as the tag’s ground plane. At angles deviating from perpendicular incidence, this effect is absent, since in specular reflection from the ground plane, the reflected wave is radiated in a different direction than the wave re-emitted by the retroactive response. This can be seen in Figure 9b, where the curve for a metal plate shows a very small RCS value related to some residual scattering from the edges of the plate. For the cross-polarized arrays, this effect of destructive interference did not occur because only cross-polarized (i.e., re-emitted) signals are received when the reflection is co-polarized, and thus, they not received by the interrogator. This means that the re-radiating mode is dominant. This is a key property of cross-polarized arrays and allows for their detection in the reflective environment, which was demonstrated in the subsequent measurements.

### 4.2. Reflective Environment Results

**Figure 13:**
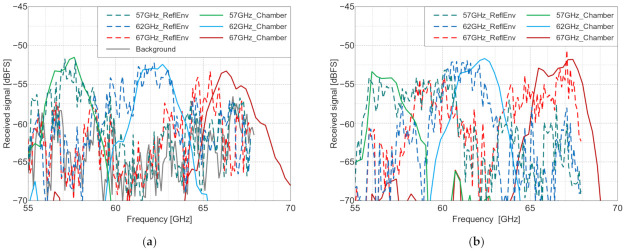
The measured received signal level in the NI measurement setup with the cross-polarized arrays placed on a door, vs. the frequency, at angles of incidence of: (**a**) 0° and (**b**) 30°. ReflEnv—measurement in the reflective environment and Chamber—normalized anechoic chamber measurement results for visual reference. The resonance frequencies of the three manufactured Van Atta tags (in GHz) are 57, 62 and 67.

**Figure 14:**
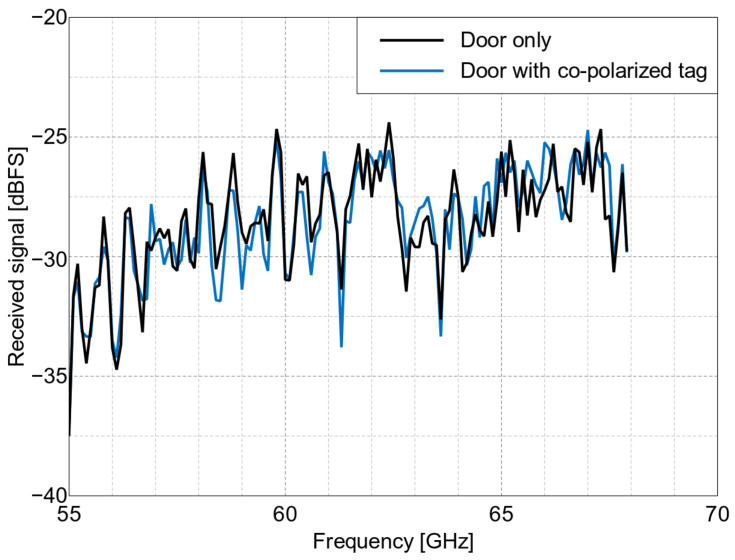
The measured received signal level in the NI measurement setup with the co-polarized array placed on a door, vs. frequency, at a 0° angle of incidence.

The non-calibrated received signal level (in dBFS—decibels relative to full scale of the receiver) measured in the setup presented in Figure 8 is plotted in Figure 13 and Figure 14. In the cross-polarized case, there are visible reflections from the environment that distort the shape of the frequency characteristics, but it was still possible to detect and distinguish the three different tags for both of the considered incident wave angles. The signal-to-background ratio, defined as the difference between the signal level of the tag’s reflection and the signal level of the background’s reflection itself (measured in the absence of the tag) at the resonance frequencies of the tags, was 7–15 dB, depending on the tag. Quite oppositely, the co-polarized array’s response was indistinguishable from the surroundings, as seen in Figure 14, and thus it cannot be straightforwardly used as an RFID tag in reflective environments. On the other hand, the co-polarized tags retain their advantage concerning RCS values and the resulting read range in use cases where the background reflections are weak.

## 5. Conclusions

In the paper, the design of co- and cross-polarized Van Atta arrays operating in V-band for MMID applications is presented. The RFID tag designs based on Van Atta arrays in the 50–70 GHz range were manufactured using cost-efficient PCB technology and are herein compared to each other. Measurement verification of the proposed tags in an experimental MMID system was performed in an anechoic chamber, as well as in a reflective environment. The measurements were carried out with both VNA and the simple NI millimeter-wave transceiver system. We compared the angular and frequency characteristics of the fabricated co- and cross-polarized tags. We have shown that in short (0.8 m) and medium (3 m) ranges, it is possible to accurately detect and distinguish a cross-polarized tag from the environment and other tags, relying solely on their amplitude response, proving that the reading is possible with a very simple transceiver system and paving the way for future commercial applications that rely on inexpensive hand-held MMID readers. The proposed approach to develop millimeter-wave chipless and batteryless tags using Van Atta arrays that extend the read distance in a wide angular range was demonstrated in a simple proof-of-concept system. It opens up new application areas as such tags can easily be integrated with road or factory infrastructure to support autonomous vehicle navigation. However, the designed tags have wide resonances, making it difficult to encode multiple bits in a narrow frequency band. Future work can be directed at decreasing the bandwidth of the resonances, thus enabling multi-bit operation. In addition, the possible applications of the tags can be extended by embedding additional functionality, e.g., sensing or combining the interrogators with radar. Novel methods for reading the presented tags can also be investigated.

## Figures and Tables

**Table 1:** Co-polarized tag dimensions.

Dimension		Value	Description
*λ*_0_	at 57 GHz	5.263 mm	Wavelength in free space
	at 62 GHz	4.839 mm	
	at 67 GHz	4.478 mm	
*λ_g_*		0.7522 *λ*_0_	Guided wavelength
*D*		0.405 *λ_g_*	Size of a patch antenna
*Lx*		0.5 *λ*_0_	Spacing of linear antenna arrays
*Ly*		*λ_g_*	Spacing of microstrip patches in a linear array
*L*1		Λ*_g_*	Length of the first interconnecting line
*Ln*		(2*n* − 1) *λ_g_*	Length of the *n*-th interconnecting line, *n* = 1, …, 4
*W*		0.30 mm	Width of the microstrip line
*Wtag*		24/22/20 mm	Width of the tag (57/62/67 GHz)
*Ltag*		21/20/19 mm	Length of the tag (57/62/67 GHz)

**Table 2:** Cross-polarized tag dimensions.

Dimension		Value	Description
*λ*_0_	at 57 GHz	5.263 mm	Wavelength in free space
	at 62 GHz	4.839 mm	
	at 67 GHz	4.478 mm	
*λ_g_*		0.7522 *λ*_0_	Guided wavelength
*D*		0.427 *λ_g_*	Size of a patch antenna
*Lx*		*λ*_0_	Spacing of linear antenna arrays
*Ly*		*λ_g_*	Spacing of microstrip patches in a linear array
*Lv*		0.446 *λ_g_*	Vertical feed microstrip line length
*Lh*		0.497 *λ_g_*	Horizontal feed microstrip line length
*Ltn*		2 *λ_g_* + 3(*n* − 1) *λ_g_*	Top interconnecting lines length, *n* = 1, …, 4
*Lbn*		2.45 *λ_g_* + 3(*n* − 1) *λ_g_*	Bottom interconnecting lines length, *n* = 1, …, 3
*W*		0.195 mm	Microstrip patch feed line width
*Wv*		0.150 mm	Vertical feed microstrip line width
*Wh*		0.50 mm	Horizontal feed microstrip line width
*Wtag*		40/36/33 mm	Width of the tag (57/62/67 GHz)
*Ltag*		25/23/21 mm	Length of the tag (57/62/67 GHz)

**Table 3:** Performance comparison of the measured co- and cross-polarized Van Atta array tags.

Parameter	Unit	Tag Type	57 GHz	62 GHz	67 GHz	Description
RCS_avg_	[dBsm]	Co-polarized	−18	−16	−15	Average RCS value in the −20° to 20° range at RCS_f0_
		Cross-polarized	−20	−21	−23	
RCS_3dB_ang_	[°]	Co-polarized	78	76	76	Angular range of −3 dB RCS level (RCS_avg_ is the reference level)
		Cross-polarized	88	104	101	
RCS_f0_	[GHz]	Co-polarized	57.08	62.26	67.3	Frequency of the maximum RCS_avg_
		Cross-polarized	56.52	61.84	66.75	
RCS_3dB_BW_ at 0° angle	[GHz]	Co-polarized	1.3	1.0	1.0	−3 dB RCS level frequency bandwidth *
		Cross-polarized	2.0	2.46	2.20	
RCS_3dB_BW_ at 30° angle	[GHz]	Co-polarized	1.0	1.1	1.0	−3 dB RCS level frequency bandwidth
		Cross-polarized	1.8	2.40	2.80	

* For the co-polarized tags, at an angle of incidence of 0°, the 3 dB RCS bandwidth is defined in reference to the average RCS level out of the resonance valley.

**Table 4:** Comparison of the presented Van Atta array tags with state-of-the-art chipless RFID tags.

Ref.	[3]	[5]	[6]	[9]	[10]	[13]	This Work
Type	Two antennas plus multi-resonator	Two antennas plus multi-resonator	Two antennas plus multi-resonator	Antenna loaded with resonators	Multiple resonators	Van Atta array-based tag	Van Atta array-based tag
Degree of realization	Table-top results	Anechoic chamber results	Simulation only	Table-top results	Table-top results	Anechoic chamber and reflective environment results	Anechoic chamber and reflective environment results
Shape	Circular patch antenna with cascaded spiral resonators	Circular patch antenna with cascaded spiral resonators	Circular patch antenna with cascaded spiral resonators	Four rectangular metallic patches loaded with multiple slot resonators	Multiple quarter-wavelength coplanar strip-line resonators	Five interconnected 1 × 5 linear cross-polarized rectangular microstrip patch arrays	Seven interconnected 1 × 4 linear cross-polarized rectangular microstrip patch arrays
Frequency range	2.35–2.55 GHz	1.9–2.6 GHz	24–37 GHz	6.5–13 GHz	2–4 GHz	27–32 GHz	57–67 GHz
Reading range	0.105–0.13 m	0.05–0.40 m	0.01 m	0.05–0.1 m	0.65 m	3 m (anechoic chamber) and 30 m (corridor with very high-gain antennas and amplifiers)	0.8 m (anechoic chamber) and 3 m (laboratory room with high-gain antennas and no amplifiers)
Modulation	Amplitude only	Amplitude and phase	Amplitude and phase	Amplitude and phase	Amplitude only	Amplitude and phase	Amplitude only
RCS	Approximately −37 dBsm (calculated from received power)	No data	Approximately −37 dBsm (calculated from received power)	Approximately −40 to −35 dBsm	Approximately −40 to −25 dBsm	Approximately −30 to −25 dBsm	Approximately −23 to −15 dBsm
Reading angular range	No data	No data	No data	No data	No data	Approximately 70–100 degrees	Approximately 70–100 degrees
Size	Approximately 200 mm × 100 mm	88 mm × 65 mm	12 mm × 7.7 mm	Approximately 20 mm × 10 and 20 mm × 20 mm	55 mm × 33 mm	Approximately 60 mm × 60 mm	Up to 25 mm × 40 mm

## Data Availability

Not applicable.
